# Antibacterial properties of *Allium sativum* L. against the most emerging multidrug-resistant bacteria and its synergy with antibiotics

**DOI:** 10.1007/s00203-021-02248-z

**Published:** 2021-02-27

**Authors:** Agnieszka Magryś, Alina Olender, Dorota Tchórzewska

**Affiliations:** 1 Chair and Department of Medical Microbiology, Medical University of Lublin, Chodźki 1 Street, (Collegium Universum), 20-093 Lublin, Poland; 2 Department of Cell Biology, Maria Curie-Skłodowska University, Akademicka 19 Street, 20-033 Lublin, Poland

**Keywords:** *Allium sativum*, Fresh garlic extract, Multidrug-resistant bacteria, Synergy, Emerging bacterial pathogens

## Abstract

Garlic has long been known as the most effective plant species in treatment of bacterial infections. Considering the vast potential of garlic as a source of antimicrobial drugs, this study is aimed to evaluate the antibacterial activity of *Allium sativum* extracts and their interactions with selected antibiotics against drug-sensitive and multidrug-resistant isolates of emerging bacterial pathogens that are frequently found in healthcare settings. As shown by the in vitro data obtained in this study, the whole *Allium sativum* extract inhibited the growth of a broad range of bacteria, including multidrug-resistant strains with bactericidal or bacteriostatic effects. Depending on the organism, the susceptibility to fresh garlic extract was comparable to the conventional antibiotic gentamycin. Since the combinations of fresh garlic extract with gentamycin and ciprofloxacin inhibited both the drug sensitive and MDR bacteria, in most cases showing a synergistic or insignificant relationship, the potential use of such combinations may be beneficial, especially in inhibiting drug-resistant pathogens. The study results indicate the possibility of using garlic as e.g. a supplement used during antibiotic therapy, which may increase the effectiveness of gentamicin and ciprofloxacin.

## Introduction

*Allium sativum* (garlic) has been grown on a massive scale on all continents for thousands of years. The species is one of the oldest vegetables cultivated by man not only for its specific taste and smell but also for its therapeutic properties. The presence of many biologically active substances in garlic, especially in its underground bulb, makes the plant suitable for use in alternative medicine. Additionally, due to the new challenges facing contemporary medicine, the therapeutic activity of the species is being studied and the plant is increasingly being applied in treatment (Bongiorno et al. 2008; Block 2010).

The properties of garlic are associated with its extremely rich composition. It contains approximately 33 sulfur compounds, 17 amino acids, enzymes, mineral salts (e.g. germanium, selenium, phosphates, calcium, and iron salts), vitamins (e.g. ascorbic acid, riboflavin, niacin, thiamine, folic acid), and valuable essential oils (Block 1985, 2010; Morioka et al. 1993; Lawson 1998; Baruchin et al. 2001; Josling 2005; Ariga and Seki 2006; Najda et al. 2016; Tchórzewska et al. 2017). Garlic is estimated to contain over two hundred chemical substances that can protect the human organism against various diseases (Gebreyohannes and Gebreyohannes 2013).

To date, garlic extract has been shown to exert potent therapeutic effects in the treatment of cardiovascular diseases, e.g. hypertension and high serum cholesterol levels (McMahon and Vargas 1993; Silagy and Neil 1994; Yeh et al. 2006; Capraz et al. 2006). Moreover, studies conducted in a rabbit model have demonstrated that long-term administration of garlic extract reduces atherosclerotic plaque (Bordia 1981). Additionally, garlic components have been reported to lower the level of fibrin in blood and can thus prevent myocardial infarction (Ernst 1994; Fukao et al. 2007).

Investigations of the antiviral properties of the plant indicate that the garlic extract is effective in the treatment of e.g. herpes simplex virus types 1 and 2, coxsackie virus species, influenza B, para-influenza virus type 3, vaccinia virus, vesicular stomatitis virus, human immunodeficiency virus type 1, and human rhinovirus type 2 (Tsai et al. 1985; Josling 2001). Additionally, compounds contained in garlic stimulate the human immune system by enhancement of macrophage activity; hence, they can be used as supportive therapy of such diseases as HIV/AIDS (Abdullah et al. 1988; Josling 2001).

Garlic extracts have also been used in research on diabetes, which confirmed its effectiveness in reduction of blood glucose levels (Ohaeri 2001; Eidi et al. 2006). Highly interesting are the reports on the role of garlic in the prophylaxis and treatment of cancer, in particular esophageal, gastric, colorectal (Steinmetz et al. 1994; Dorant et al. 1996; Kainsa et al. 2012; Galeone et al. 2006), liver (Kweon et al. 2003), and prostate cancer (Hsing et al. 2002; Mehraban et al. 2006). Excellent results have also been reported in the case of topical treatment of fungal diseases with garlic extract (Sabitha et al. 2005; Ledezma and Apitz-Castro 2006) and intravenous administration of such extract (Lemar et al. 2007). Garlic has long been recommended as an effective remedy for intestinal parasites (Kalyesa et al. 1975; Mirelman et al. 1987; Ankri et al. 1997). Additionally, by increasing the serum levels of such enzymes as catalase and glutathione peroxidase, it serves as an efficient antioxidant (Prasad et al. 1995). Various authors describing the health-enhancing properties of garlic also indicate its role in prevention of cognitive function decline (Borek 2006). Garlic has also been found to protect against the negative effects of stress, as demonstrated by studies on animals subjected to physical and mental pressure (Kasuga et al. 1999; Morihara et al. 2006; Nance et al. 2006).

Garlic has long been known as the most effective plant species in treatment of bacterial infections (Subramanyan et al. 1958). Extract from the underground garlic bulb, especially its compound allicin (diallyl-thiosulfinate), inhibits the growth of many species of Gram-positive and Gram-negative bacteria (Yoshida et al. 1999; Tsao and Yin 2001; Bakri and Douglas 2005). Therefore, garlic is an excellent alternative to the antibiotics used currently. Furthermore, researchers are attempting to find new drugs due to the recent emergence of bacterial strains with resistance to traditional antibiotics. One of the solutions to this problem seems to be to develop a combination of garlic extracts and antibiotics available on the market with partial or complete synergism between the substances (Didry et al. 1992; Jonkers et al. 1999).

For the last few decades, the incidence of microbial infections caused by multidrug-resistant pathogens has increased dramatically. Multidrug resistance (MDR), defined as the ability of a microorganism to resist the action of an administered antimicrobial drug despite earlier sensitivity to the substance, has become a significant public health threat, as almost all antimicrobial agents available are subject to the problem (Jyoti et al. 2014; Magiorakos et al. 2011; Gaekwad and Trivedi 2013). Although antimicrobial resistance occurs naturally over time, extensive spread of MDR pathogens, especially in immunocompromised patients, is almost always associated with poor prognosis (Asokan et al. 2019; Magiorakos 2011).

Drug resistance is detected in both Gram-positive and Gram-negative bacteria. In 2017, WHO published a list outlining the global priority for research and development of new antibiotics. Among these, highly resistant *Enterobacteriaceae* with *Escherichia coli* and *Klebsiella pneumoniae* (carbapenem-resistant and 3rd generation cephalosporin-resistant) and *Pseudomonas aeruginosa* (carbapenem-resistant) were at the top of the list as critical and urgent threats that require greater global attention and swifter action (Brunel and Guery 2017; Asokan et al. 2019; Jyoti et al. 2014). Methicillin-resistant *Staphylococcus aureus* (MRSA) and vancomycin-resistant *Enterococcus* (VRE) were also mentioned by the Organization and classified as high priority pathogens (Brunel and Guery 2017; Asokan et al. 2019; Jyoti et al. 2014). Besides being multidrug-resistant, these bacteria are also common hospital-acquired pathogens.

Therefore, in light of the evidence of the global spread of multidrug-resistant pathogens, the need to find an alternative strategy to antibiotic treatment is of paramount importance (Brunel and Guery 2017; Manandhar et al. 2019). One of the solutions to this problem seems to be to potentiate the effect of existing antibiotics through combination therapy.

Considering the vast potential of garlic as a source of antimicrobial drugs, this study goal is to evaluate the antibacterial activity of *Allium sativum* extracts and their interactions with selected antibiotics against drug-sensitive and multidrug-resistant isolates of emerging bacterial pathogens that are frequently found in healthcare settings (*Staphylococcus aureus*, *Enterococcus faecalis*, *Escherichia coli*, *Pseudomonas aeruginosa*, and *Klebsiella pneumoniae*).

## Materials and methods

### Plant material

Garlic (*Allium sativum* cultivar Arkus) obtained from Krakow Horticulture and Seed Production POLAN was grown in the Botanical Garden of Maria Curie-Skłodowska University in Lublin. The Botanical Garden of Lublin is situated in the NW part of the town at latitude 51°16ʹ N and longitude 22°30ʹ E. Brown soils prevail in the area (Index Seminum 2014 Hortus Botanicus Universitatis Marie Curie-Skłodowska). *A. sativum* bulbs were planted in autumn (October) 2018. Plant harvest was carried out in August 2019. All agrotechnical practices applied to the field during plant vegetation followed the recommendations developed for garlic. No herbicides, fungicides, or any chemical agents were introduced during cultivation, and manual weed control methods were used. Macroscopic images were taken with a Nikon D300 camera equipped with an AF MICRO NIKKOR 60 mm objective.

### Preparation of fresh garlic extract

Fresh garlic (*A. sativum* cv. Arkus) bulbs were peeled, weighed (24 g), and washed with water first and then soaked in 70% ethanol. The ethanol was allowed to evaporate in a sterile laminar flow chamber, and the garlic was homogenized aseptically using a sterile mortar and pestle. The extract was centrifuged at 6000 rpm for 10 min, and the supernatant was filtered through a 0.45-μm membrane.

The concentration used in this study is the total weight of garlic per ml. Twenty-four grams of raw garlic yielded 4 ml of extract (i.e. 6 g/ml). This extract was regarded as the 100% concentration. Each time, the 100% garlic extract was inoculated on nutrient agar media and incubated at 37 °C overnight for sterility.

### Bacterial strains used

Four drug-sensitive reference strains consisting of *Enterococcus faecalis* (ATCC 29212), methicillin-susceptible *Staphylococcus aureus* (MSSA, ATCC 29213), *Pseudomonas aeruginosa* (ATCC 27853), and *Escherichia coli (*ATCC 25922) and four multidrug-resistant strains of vancomycin-resistant + high level aminoglycoside-resistant *Enterococcus faecalis* (VRE + HLAR, ATCC 51299), metallo-β-lactamase *Escherichia coli* (MBL, ATCC 35218), methicillin-resistant *Staphylococcus aureus* (MRSA, NTC 12493), and extended β-lactamase *Klebsiella pneumoniae* (ESBL, ATCC 700603) were used in this study.

All bacterial strains were grown on Mueller–Hinton Agar (MHA) at 37 °C.

### Well Diffusion Method

For well diffusion, bacterial suspensions (MacFarland = 0.5) were inoculated on MHA plates using a sterile cotton swab. The plates were allowed to dry for 15 min. and wells with a 6-mm diameter were punched out using a cork borer. Each well was filled with 50 μl of 100% garlic extract (v/v). A gentamicin disc (30 μg/ml) was used as a reference antibiotic against bacterial species (positive control).

The plates were incubated for 24 h. The diameter of the inhibition zone was measured, and the plates were photographed.

### Determination of minimum inhibitory concentration (MIC) and minimum bactericidal (MBC) concentration

Susceptibility testing was performed following the European Committee on Antimicrobial Susceptibility Testing (EUCAST) guidelines using the broth dilution method in a 96-well microtiter plate format as previously described (Leontiev et al. 2018). Bacterial colonies from the MHA plates incubated overnight at 37 °C were resuspended in sterile NaCl and adjusted to the 0.5 McFarland standard of approximately 1.2 × 10^8^ CFU/ml.

For broth microdilution, susceptibility panels in 96-well microtiter plates were prepared by dispensing 100 μl of the garlic solutions with the highest concentrations (100%) into the first column wells and 50 μl of MHB (pH 5.9) into the other wells. Then, two-fold serial dilutions of the garlic solutions were made by drawing up 50 μl of the garlic solution in the first column wells into the second column and then moving on to the next column to achieve the final concentrations. Aliquots (50 μl) of each bacterial suspension were inoculated into the wells of the microtiter plates to obtain a final volume of 100 μl in each well of the plate. The last two wells were positive and negative controls, respectively. The positive control was inoculated with bacterial suspension only, while the negative well was left blank without inoculation. The 96-microwell plates were sealed using a perforated plate seal and incubated at 37 °C for 24 h. The MICs of the garlic extracts were recorded as the lowest concentration where no viability was observed in the wells of the 96-microwell plates after incubation for 24 h. After incubation, 40 μl of a 0.2 mg/ml aqueous solution of methylthiazoyltetrazolium chloride (MTT) were added to each well and further incubated for 30 min at room temperature. MIC was defined as the lowest concentration in which no transformation of MTT was observed. Gentamicin, to which the test strains were sensitive, was used as a positive control, and its MIC was determined using the same procedure. All samples were tested in triplicate and the tests were repeated twice.

To determine the minimal bactericidal concentration, 10 μl out of each well were inoculated onto the MHB plate and incubated for 24 h. The lowest concentrations that showed no growth after 24 h gave the MBC value.

### Growth kinetics

The fresh garlic extract was serially diluted in the MHB medium. The overnight bacterial cultures were adjusted to MacFarland = 0.5 and mixed with each dilution in the 96-well plate. Growth was monitored in a plate reader at 600 nm for 24 h at 37 °C.

### Checkerboard method for the combination

The antimicrobial effect of combinations consisting of FGE with gentamicin or ciprofloxacin was assessed with the checkerboard method. Serial dilutions of antibiotics to at least double the MIC were prepared according to the recommendations of EUCAST immediately prior to testing and mixed in a 96-well plate containing varying concentrations of FGE (6–0.005 g/ml) in CSMHB. Each well was inoculated with 100 μl of the bacterial inoculum of 5 × 10^5^ CFU/ml, and the plates were incubated at 35 °C for 24 h.

To validate the MIC, the fractional inhibitory concentration (FIC) was calculated. The following formulas were used to calculate the FIC index: FIC_FGE_ = (MIC_FGE_ in combination)/(MIC_FGE_ alone), FIC_antibiotic_ = (MIC_antibiotic_ in combination)/(MIC_antibiotic_ alone), and the FIC index = FIC_FGE_ + FIC_antibiotic._ The FIC indices were used to characterize the antibiotic interactions as follows: synergy: when the combination of compounds results in a FIC value of < 0.5, it increases the inhibitory activity (decrease in MIC) of one or both compounds in comparison with the compounds alone. Additivity or indifference: when the combination of compounds results in a FIC value of 0.5–4, there is no increase in inhibitory activity or a slight increase in inhibitory activity from the additive effect of both compounds combined. Antagonism: when the combination of compounds results in a FIC value of > 4, it increases the MIC or lowers the activity of the compounds (Pillai et al. 2013).

### Statistical analysis

Statistical analyses were performed using a 2-tailed unpaired *t*-test (2 groups) or one-way ANOVA followed by Turkey’s multiple comparisons post-hoc test. *p* < 0.05 was considered statistically significant. All data are expressed as the mean ± SD in the text.

## Results

### Morphological analysis of garlic

**Fig. 1:**
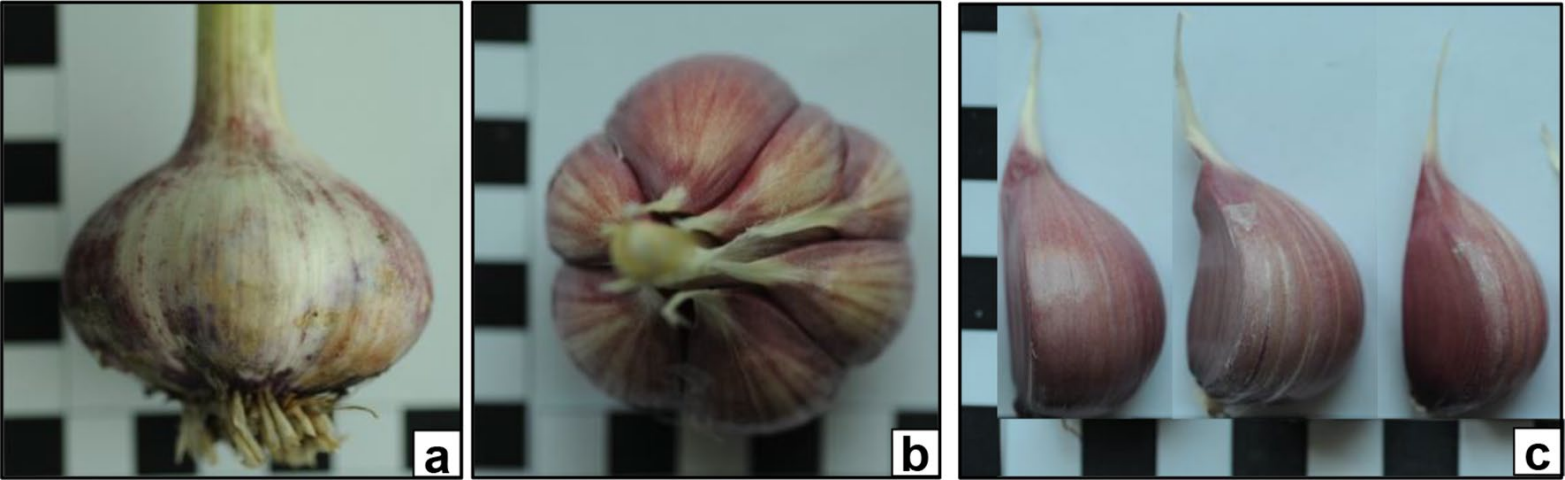
Morphology of the underground bulb of *Allium sativum* (**a**). Bulb devoid of the cover with purple cloves (**b**). Single cloves (**c**)

The underground part of *A. sativum* cv. Arkus is a bulb, which is covered with scaly purple leaves and has a flat shape of the base. The diameter of the bulb is approximately 5 cm (Fig. 1a). It consists of cloves covered with light purple scales and arranged radially within the bulb (Fig. 1b). The number of cloves in the bulb ranges from 5 to 8 and their size is from 3 cm to 1.5 cm (Fig. 1c).

### Antimicrobial effects of fresh garlic extract using a well-diffusion method

**Fig. 2:**
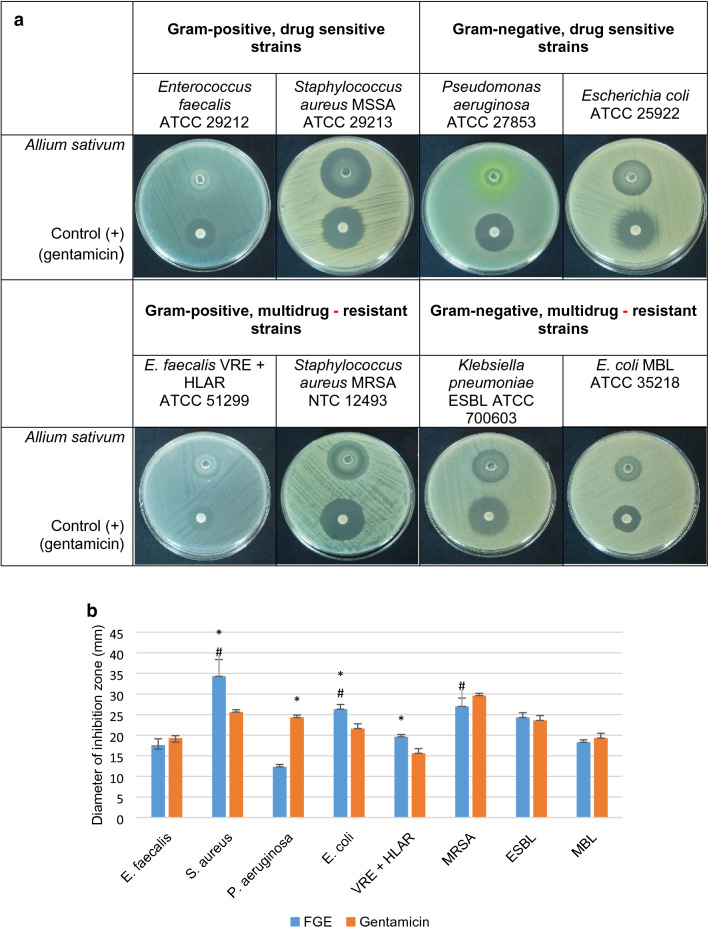
Antimicrobial activity of *A. sativum* fresh extract against drug-sensitive and multidrug-resistant strains of bacteria determined with the well-diffusion method. **a** representative pictures of the studied bacteria. Standardized bacterial inoculum was spread evenly on the MHA plate to yield a lawn culture. The upper well contains 50 μl of FGE (100%), and the lower part is a filter disc containing 30 μg/ml gentamicin (commercially available). **b** zones of inhibition of fresh garlic extract and gentamicin (control +) for the tested bacterial strains. The data represent mean ± SD (*n* = 4). *p < 0.05 FGE vs. gentamicin (*t*-test); ^#^*p* < 0.05 FGE results between bacteria tested (ANOVA test)

The antimicrobial potential of the 100% fresh garlic extract was evaluated by measuring the zones of inhibition of the tested bacterial pathogens, and the results (zone of inhibition) were compared between the pathogens and with the control (gentamicin 30 μg/ml). Initial screening revealed that FGE exhibited antibacterial activity against all the tested organisms, with a variable degree of their sensitivity. Of all the bacteria tested, FGE was the most effective against *S. aureus MSSA, E. coli*, and *S. aureus* MRSA (*p* < 0.05) (Fig. 2a, b).

Considering antibacterial activity, FGE was significantly more effective against *S. aureus*, *E. coli*, and *E. faecalis* VRE + HLAR than the control antibiotic used (gentamicin) (*p* < 0.005). Among the tested bacteria, *P. aeruginosa* was the least susceptible to FGE, compared to gentamicin and the other tested bacteria in this study (*p* < 0.05). Moreover, there were no significant differences between the antibacterial activity of *Allium sativum* extract and the control antibiotic in the case of three of the four isolates with confirmed resistance mechanisms, i.e. *S. aureus* MRSA, *K. pneumoniae* ESBL, and *E. coli* MBL (Fig. 2a, b).

### Determination of MIC and MBC values of fresh garlic extract

The information whether the tested antibacterial agent has bacteriostatic/bactericidal potential in vitro is important, as it relates to the activity of the new drug and monitors the level of resistance of the microorganisms to the tested agent (Pankey and Sabath 2004). Therefore, the MIC and MBC values of FGE for the tested bacterial pathogens were determined in the next step.

As indicated in Table 1, the fresh *A. sativum* extract had the lowest MIC value of 6.25% against 2 drug-sensitive bacteria: *S.* *aureus* MSSA and *E. coli* and 3 multidrug-resistant pathogens: *S. aureus* MRSA, *K. pneumoniae* ESBL, and *E. coli* MBL. The highest MIC value of 50% was noted for *E. faecalis* VRE + HLAR, whereas the growth of its drug-sensitive counterpart was inhibited at the concentration of 25%.

**Table 1:** MIC and MBC values (% v/v) of fresh garlic extract for different bacterial strains.

	Drug sensitive strains				Multi drug-resistant strains			
	*E. faecalis*	*S. aureus MSSA*	*P. aeruginosa*	*E. coli*	*E. faecalis* VRE + HLAR	*S. aureus* MRSA	*K. pneumoniae* ESBL	*E. coli* MBL
MIC FGE % (mg/ml)	25 (1500)	6.25 (375)	12.5 (750)	6.25 (375)	50 (3000)	6.25 (375)	6.25 (375)	6.25 (375)
MBC FGE % (mg/ml)	–	25 (1500)	12.5 (750)	6.25 (375)	–	50 (3000)	12.5 (750)	12.5 (750)

Minimum inhibitory concentration (MIC)—the lowest concentration of FGE that inhibits bacterial growth. Minimum bactericidal concentration (MBC)—the lowest concentration of FGE that kills bacteria. The table shows the percentage of FGE (v/v) that inhibited (MIC) or killed (MBC) the tested organisms. The values are given as the highest value out of two replicates

It was also found that the MBC value of FGE against a majority of the analyzed microorganisms was at least two-fold higher than the corresponding MIC. The MIC and MBC of the tested bacteria were equal in the case of *P. aeruginosa* and *E. coli,* with a mean value of 12.5% and 6.25% respectively, in both assays. The fresh garlic extract exerted a bactericidal effect against most of the bacteria, including *S. aureus* MSSA, *P. aeruginosa, E. coli*, and MDR bacteria with *K. pneumoniae* ESBL and *E. coli* MBL patterns of resistance. *Staphylococcus aureus* MRSA was less susceptible to the bactericidal effects of garlic than the other bacteria tested.

### Effects of FGE on the bacterial growth dynamics

**Fig. 3:**
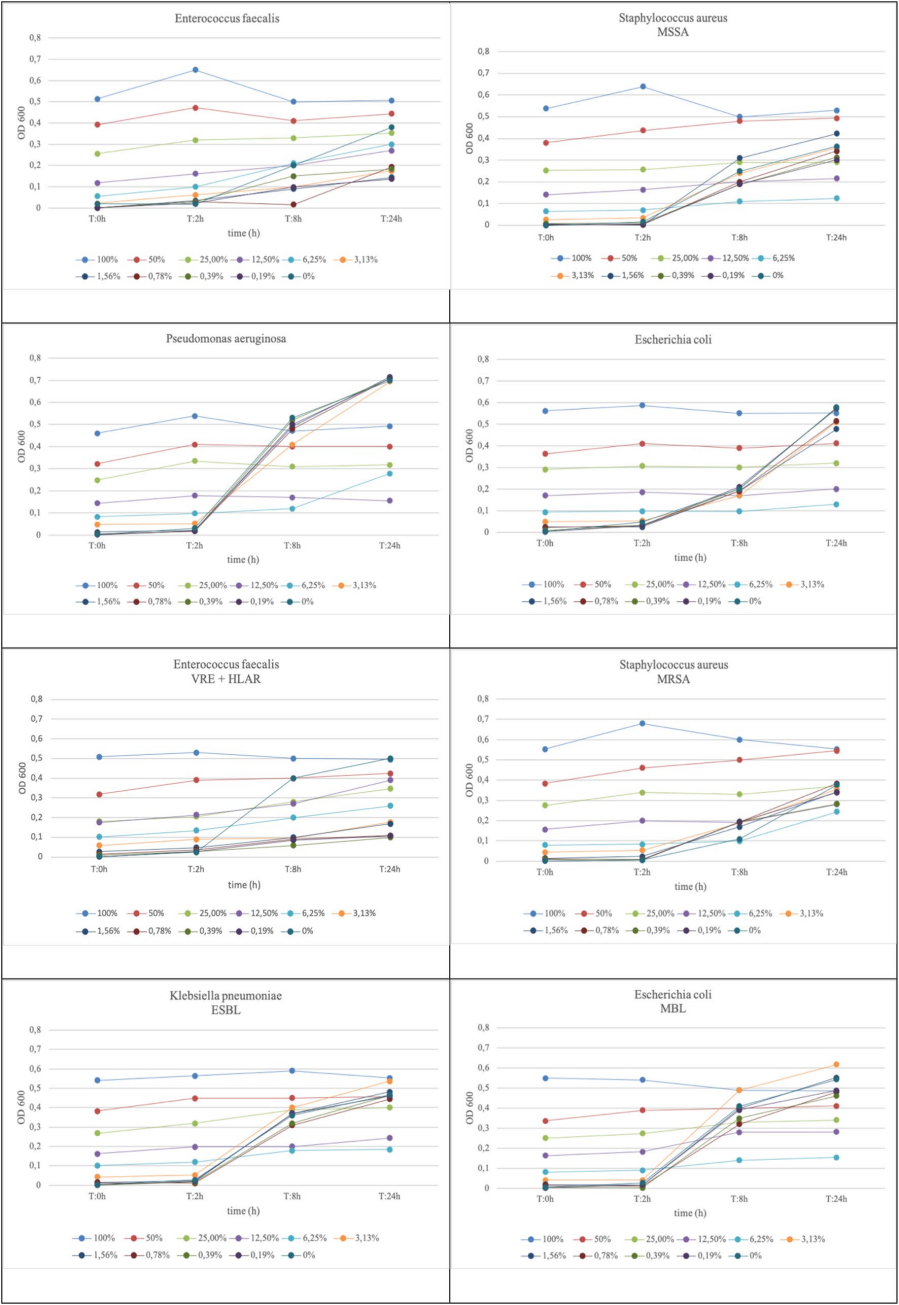
Effects of different concentrations (0%–100%) of FGE on the bacterial growth determined after 0 h, 2 h, 8 h, and 24 h of exposure. The Figure shows a representative plot chosen from 3 replicates

In contrast to the MIC and MBC tests, which provide only end-point results of drug susceptibilities, the monitoring of the bacterial growth kinetics over time at different concentrations of FGE revealed the relative sensitivity of the bacteria to the tested compound. Therefore, in the next experiment, all bacteria were exposed to decreasing concentrations of FGE (100%–0%) over a 24-h period. In all cases, FGE exerted concentration-dependent effects on basal microbial growth dynamics (Fig. 3). In agreement with our previous observations, the MIC values of FGE coincided with the final data sets of growth kinetic for corresponding bacteria.

*Staphylococcus aureus* and *E. coli* exposed to the 100%-6.25% concentrations of FGE showed no increase in OD over 24 h. However, at the lower concentrations of the extract, the cells began to grow exponentially at a rate comparable to that of the garlic-free medium.

After a ca. 2-h lag phase, the *P. aeruginosa* culture containing FGE at the concentration of 12.5% showed a sharp increase in OD, reaching values similar to the control culture.

The fresh garlic extract at the high concentrations (100%–25%) caused inhibition of *E. faecalis*, for which growth was observed at the concentrations of 12.5% and lower. However, it was unable to reach the final OD value of the control culture without the extract.

An extended lag phase with a maximum duration of > 8 h was observed in *E. faecalis* VRE + HLAR exposed to FGE, irrespective of the extract concentration. The culture exposed to the *A. sativum* extract concentration of 25% and less began to grow exponentially, but the final OD values did not reach that of the control. The exponential growth of the other MDR bacteria tested was observed in cultures exposed to FGE of < 6.25% reaching OD values comparable to that of the extract-free control medium.

### Interaction between fresh garlic extract and antibiotics assessed with the checkerboard method

Combination therapies of antibiotics and antimicrobial plant extracts provide an alternative strategy for treating infectious diseases. Synergy, if demonstrated, is indicated by an increase in the the inhibitory activity and a successful combination of the compounds. Therefore, in the next step, the checkerboard method was used to determine the interaction between FGE and two antimicrobial drugs: gentamicin and ciprofloxacin in the test strains (Table 2).

**Table 2:** Antimicrobial effect of combinations consisting of FGE (drug A) and selected antibiotics (drug B) against the tested bacterial strains assessed with the checkerboard method

Bacterial strain	FGE—GM			FGE—CIP		
	Ratio	ΣFIC	Activity	Ratio	ΣFIC	Activity
*E. faecalis*	0.03:0.25	0.28	S	1:0.5	1.5	I
*S. aureus* MSSA	0.029:0.124	0.15	S	0.12:0.124	0.24	S
*P. aeruginosa*	0.026:2	2.02	I	0.249:0.12	0.36	S
*E. coli*	0.1:0.062	0.16	S	0.4:2	2.4	I
*E. faecalis* VRE + HLAR	0.48:2	2.48	I	2:4	6	A
*S. aureus* MRSA	0.13:0.3	0.4	S	0.4:0.5	0.9	I
*K. pneumoniae* ESBL	0.24:0.125	0.36	S	0.24:0.125	0.36	S
*E. coli* MBL	1.05:3	4.05	A	2:1	3	I

Based on the FIC index, the antibacterial activity of gentamicin was highly influenced by the combination with FGE against the drug-sensitive bacteria, except *P. aeruginosa*, for which the combination exerted an indifferent effect. Similarly, in *S. aureus* MRSA and *K. pneumoniae* ESBL, the combination of FGE and gentamicin showed improved activity, making the drug-resistant bacteria treatable with the clinically recommended dosage. The combination gave an antagonistic effect in one case, i.e. *E. coli* MBL.

The combination of FGE with ciprofloxacin yielded a synergistic interaction in *S. aureus* MSSA*, P. aeruginosa*, and *K. pneumoniae* ESBL—a drug-resistant bacterium. For *E. faecalis*, *E. coli*, *S. aureus* MRSA, and *E. coli* MBL, such a combination yielded no increase in inhibitory activity or a slight increase in inhibitory activity from the additive effect of both compounds combined, as the FIC values ranged from 0.9 to 3. The combination regimen of the compounds increased the MIC for *E. faecalis* VRE + HLAR, contributing to a decrease in the activity of a ciprofloxacin.

## Discussion

Currently, antibiotics are widely used worldwide both in medicine and in agriculture, e.g. animal husbandry. It seems that such a widespread use of antibiotics has contributed to the emergence of drug-resistant bacteria, which is one of the greatest threats to humans. This phenomenon has been observed in both Gram-positive and Gram-negative bacteria. This is a major social problem combined with the reduced immunity of the human organism exposed to the huge negative pressure of the environment. Hence, the current search for new alternative therapies based on plant resources seems promising. *A. sativum* analyzed in this study is extremely rich in biologically active substances with multifarious therapeutic effects. The study was focused on determination of the antibacterial properties of garlic against multidrug-resistant reference strains of bacteria posing the greatest health threats. Additionally, interactions of garlic extract with certain antibiotics used against drug-sensitive and multidrug-resistant bacteria were analyzed.

With an increase in antibiotic resistance, the use of herbal extracts as a source of therapeutic agents is receiving considerable attention in global health debates (Bharwaj et al. 2016; Reiter et al. 2020; Bayan et al. 2014; Borlinghans et al. 2014). Previous studies have shown that allicin, i.e. one of the main antibacterial compounds of fresh garlic, significantly inhibits a variety of infectious agents (Abouelfetouh and Moussa 2012; Bhardwaj et al. 2016; Reiter et al. 2020; Bayan et al. 2014; Borlinghans et al. 2014). Although the precise interaction between allicin and bacteria has not been clarified yet, the commonly accepted mechanism involved in the bactericidal action of allicin is proposed to be due to its reaction with cysteine-containing enzymes involved in key biosynthetic pathways. Thus, many bacterial sulfhydryl enzymes are oxidized and inhibited when exposed to allicin (Leontiev et al. 2018; Bayan et al. 2014; Borlinghaus et al. 2014; Fujisawa et al. 2009). Moreover, allicin is quite lipophilic, which leads to alterations in the structure and integrity of the microbial phospholipid membranes probably accounting for cellular content leakage (Salehi et al., 2019; Leontiev et al. 2018). An important advantage of allicin contained in garlic extract over most antibiotics is that it does not target a specific protein in the bacterial cell, and therefore it appears that resistance associated with modification of the target site on it should not occur (Muller et al. 2016).

Given previous evidence that allicin has great antibacterial activity, the present study investigated the potential of using garlic for controlling multidrug-resistant pathogens with the greatest epidemiological significance. *Klebsiella pneumoniae*, *Escherichia coli*, and *Pseudomonas aeruginosa* are the most common Gram (−) organisms associated with multi-resistance. These bacteria are commonly resistant to fluoroquinolones, aminoglycosides, and β-lactams with the last-resort treatment, i.e. carbapenem antibiotics, making their infections untreatable. Additionally, management of multidrug-resistant Gram (+) bacteria, especially *Enterococci* and *Staphylococcus aureus*, is a real challenge (Brunel and Guery 2017). New or improved classes of antimicrobials with significant antimicrobial activity and lower toxicity are therefore urgently needed to overcome this threat (Rachman et al. 2017; Vu Van Loi et al. 2019).

Garlic has been shown to exhibit broad-spectrum antimicrobial activity against Gram (+) and Gram (−) bacteria (Nakamoto et al. 2019; Muller et al. 2016; Borlinghaus et al. 2014; Bharwaj et al. 2016). Consistent with the previous reports, the *Allium sativum* extract at the minimal concentration of 6.25% (375 mg/ml) successfully inhibited the growth of drug-susceptible *S. aureus* and *E. coli* and most of the drug-resistant bacteria with the MRSA, ESBL, and MBL patterns of resistance, demonstrating the maximum activity against *E. coli*, and *S. aureus* MSSA and MRSA.

The study also revealed that FGE at the 100% concentration had the same (MRSA, ESBL, MBL, *E. faecalis*) or higher (*S. aureus*,* E. coli*, VRE + HLAR) level of susceptibility as the antibiotic used (gentamycin).

Methicillin resistance means resistance to all anti-staphylococcal β-lactam antibiotics (except ceftaroline and ceftobiprole) and to other commonly used agents, including erythromycin, clindamycin, and fluoroquinolones, whereas MSSA has no antibiotic resistance. Noteworthy, both tested *S. aureus* strains were significantly susceptible to FGE. These data therefore suggest that the antibiotic-resistance mechanisms of MRSA have no impact on susceptibility to garlic. This is probably related to the fact that allicin readily penetrates Gram (+) cell wall and reacts with conserved target proteins inside the microbial cell (Fujisawa et al. 2009). Vu Van Loi et al. identified 57 proteins with *S*-thioallylations under allicin treatment that interfere with bacterial metabolic homeostasis (Van Loi et al. 2019). They also revealed that allicin caused a strong thiol-specific oxidative and sulfur stress response and protein damage in *S. aureus* (Van Loi et al. 2019). Importantly, due to multiple targets of allicin, bacteria cannot overcome the injury through mutation or metabolic adaptation (Fujisawa et al. 2009).

Of the MDR bacteria, highly resistant Gram (−) organisms, such as *Enterobacteriaceae* carrying ESBL and MBL resistance mechanisms, require special mention. These organisms are resistant to the first-choice antibiotics and especially MBLs are resistant to all currently available antimicrobial agents (Magiorakos et al. 2011). The present study demonstrated that FGE at a concentration of 375 mg/ml (6.25%) successfully inhibited the growth of the major G (−) MDR bacteria with all strains killed at 2 × MIC. The inhibitory effect of FGE against Gram (−) MDR bacteria resembled the inhibition pattern observed in the drug-susceptible strain of *E. coli*. This fact clearly indicates that any antibiotic-resistance mechanism represented by these MDR bacteria seemingly has no impact on susceptibility to garlic.

Of all the tested reference bacterial strains, *Pseudomonas aeruginosa* among Gram (−) bacteria and *E. faecalis* among Gram (+) bacteria exhibited relative garlic resistance. To achieve inhibition of growth in the exponentially growing *P. aeruginosa,* the 12.5% (750 mg/ml) concentration of FGE was required to be effective against the pathogen.

The effect of FGE against *E. faecalis* and its VRE + HLAR resistant variant was remarkably different from the other bacteria tested, as their initial lag phase of growth was extended. The pattern of the lag phase extension in these cases is likely to be caused by either the bacteriostatic properties of FGE or non-lethal cell injury. There is also a possibility that *Enterococci* thickened their cell wall in an effort to survive under the selective pressure of garlic.

With an increase in antibiotic resistance, the idea of potentiation of antimicrobial activity by combining herbal drugs and antibiotics seems to be a promising approach (Bharwaj et al. 2016). So far, the majority of investigations of the garlic/allicin-antibiotic interaction appear to be directed mostly toward β-lactam antibiotics (Cai et al. 2007; Shah and Williamson 2019; Pillai et al. 2013; Gaekwad and Trivedi 2013). The present study, however, investigated the potential of using garlic as an adjunct to gentamycin and ciprofloxacin therapy for synergistic association against multidrug-resistant pathogens. A probable explanation for the enhancement of antimicrobial activity of antibiotics when combined with garlic could be the alteration in the structure and integrity of the bio membranes facilitating the uptake and subsequent achievement of the target by the tested antibiotics (Muller et al. 2016; Abouelfetouh and Moussa 2012). It is also possible that garlic and the antibiotic used inhibit similar or related sites of action in the bacterial cell.

In this study, gentamycin was selected for the combination with FGE, as it is a common broad-spectrum antibiotic indicated for acute serious infections. When combined with gentamycin, FGE was found to enhance the antibacterial activity of the drug-susceptible *E. faecalis, S. aureus*, and *E. coli* and the drug-resistant bacteria—MRSA and ESBL. Thus, the *A. sativum* extract with gentamycin reduced the gentamycin resistance by lowering the MIC value, which indicates a positive association between the two tested antimicrobial agents.

The antagonistic effect between garlic and gentamycin was observed in the case of *E. coli* MBL. Metallo-β-lactamases are enzymes that hydrolyze carbapenems, the last-resort drugs in treatment of Gram (−) bacterial infections. Pathogenic bacteria that produce MBLs are usually resistant to virtually all antibiotics used in clinical medicine today (Ejikeugwu et al. 2017). Attempts to identify inhibitors of MBLs revealed that thiol-containing small molecules could have great inhibitory activity against these enzymes (Tehrani and Martin 2017). In our experiment, however, the exposure of MBL to the combination of the allicin-containing garlic extract with either gentamycin or ciprofloxacin failed to reduce the MICs of these drugs, indicating that their inhibitory capacity is targeted at unrelated sites.

In the present study, the interaction between garlic and ciprofloxacin was also assessed, since the drug is on the WHO List of Essential Medicines as the safest and most effective drug needed in the health system. However, many bacteria have already developed resistance to the drug in recent years (WHO 2019). In general, the combination of garlic with ciprofloxacin appeared to be less effective against the multidrug-resistant pathogens, since synergy was displayed only with *K. pneumoniae* ESBL. In turn, the treatment of the drug-sensitive strains with the ciprofloxacin-garlic extract combination proved to be synergistic in the case of *S. aureus* and *P. aeruginosa*, indicating that the use of garlic enhanced the antibacterial activity of ciprofloxacin.

## Conclusions

As shown by the in vitro data obtained in this study, the whole *Allium sativum* extract inhibited the growth of a broad range of bacteria, including MDR strains with bactericidal or bacteriostatic effects. Depending on the organism, the susceptibility to FGE was comparable to the conventional antibiotic gentamycin. Since the combinations of FGE with gentamycin and ciprofloxacin inhibited both the drug-sensitive and MDR bacteria, in most cases showing a synergistic or insignificant relationship, the potential use of such combinations may be beneficial, especially in inhibiting drug-resistant pathogens. The study results indicate the possibility of using garlic as e.g. a supplement used during antibiotic therapy, which may increase the effectiveness of gentamicin and ciprofloxacin. It can be assumed that this positive effect may also apply to other groups of antibiotics, but further research is needed.
